# A novel dynamic model for predicting outcome in patients with hepatitis B virus related acute-on-chronic liver failure

**DOI:** 10.18632/oncotarget.22447

**Published:** 2017-11-14

**Authors:** Ran Xue, Zhonghui Duan, Haixia Liu, Li Chen, Hongwei Yu, Meixin Ren, Yueke Zhu, Chenggang Jin, Tao Han, Zhiliang Gao, Qinghua Meng

**Affiliations:** ^1^ Department of Critical Care Medicine of Liver Disease, Beijing You-An Hospital, Capital Medical University, Beijing, China; ^2^ Georgia Prevention Institute, Medical College of Georgia, Augusta University, Augusta, GA, USA; ^3^ The School of Social Development and Public Policy, Beijing Normal University, Beijing, China; ^4^ Department of Hepatology, Tianjin Third Central Hospital of Tianjin Medical University, Tianjin, China; ^5^ Department of Infectious Diseases, The Third Affiliated Hospital, Zhongshan University, Guangzhou, China

**Keywords:** acute-on-chronic liver failure, hepatitis B, prognostic model

## Abstract

**Aim:**

It is challenging to predict the outcome of patients with hepatitis B virus related acute-on-chronic liver failure (HBV-ACLF) through existing prognostic models. Our aim was to establish a novel dynamic model to improve the predictive efficiency of 30-day mortality in HBV-ACLF patients.

**Methods:**

305 patients who were diagnosed as HBV-ACLF (derivation cohort, n=211; validation cohort, n=94) were included in this study. The HBV-ACLF dynamic (HBV-ACLFD) model was constructed based on the daily levels of predictive variables in 7 days after diagnosis combined with baseline risk factors by multivariate logistic regression analysis. The HBV-ACLFD model was compared with the Child-Turcotte-Pugh (CTP) score, end-stage liver disease (MELD) score, and MELD within corporation of serum sodium (MELD-Na) score by the area under the receiver-operating characteristic curves (AUROC).

**Results:**

The HBV-ACLFD model demonstrated excellent discrimination with AUROC of 0.848 in the derivation cohort and of 0.813 in the validation cohort (p=0.620). The performance of the HBV-ACLFD model appeared to be superior to MELD score, MELD-Na score and CTP score (P<0.0001).

**Conclusion:**

The HBV-ACLFD model can accurately predict 30-day mortality in patients with HBV-ACLF, which is helpful to select appropriate clinical procedures, so as to relieve the social and economic burden.

## INTRODUCTION

Acute-on-chronic liver failure (ACLF) is a life-threatening syndrome with varied etiology and manifestations with a short-term mortality of 50–90% [1]. ACLF is defined as the acute decompensation of liver function in patients with either previously diagnosed or undiagnosed chronic liver disease [2–3]. Hepatitis B virus (HBV) is the leading cause of chronic liver disease in the Asia-Pacific region, including China and India [4]. Liver transplantation (LT) is a feasible and beneficial treatment for patients with ACLF to achieve survival [5]. It is important to accurately distinguish the ACLF patients who need LT, and grasp the best opportunity for LT [6]. To guide and optimize therapeutic strategy for ACLF patients, an accurate prognostic scoring system is prerequisite [7].

Varied prognostic scoring systems have been developed to predict ACLF mortality and guide the decision-making of LT. The Child-Pugh-Turcotte (CPT) classification and the model of end-stage liver disease (MELD) score are the most commonly used for patient priority on the waiting list of LT [8–9]. MELD Serum sodium (MELD-Na) score, as a modified MELD score, presented promising value for predicting mortality among patients on the LT waiting list [10–11].

However, each of the models above cannot apply reasonably to evaluate the clinical outcome in hepatitis B virus related acute-on-chronic liver failure (HBV-ACLF), mainly due to differences in patient background queues. There exist great differences between Eastern and Western ACLF in definition and diagnostic criteria, involving the basis of chronic liver disease, the concept of organ failure and the diagnostic criteria for organ failure [12–13]. Furthermore, HBV-ACLF is a dynamic process in which the variables at the time of hospitalization are predicted to vary over time, accompanied with the clinical processes and outcomes change accordingly. The existing prognostic models were established based on static baseline, which cannot logically evaluate the predictive outcome [14]. Therefore, it is urgently needed to derivation and validation of a novel dynamic model for predicting outcome in patients with HBV-ACLF.

Our aim was to establish a prognostic model according to early changes of independent predictive variables at admission in patients with HBV-ACLF; to identify whether the model was superior to the existing prognostic models such as CPT scores, MELD scores, and MELD-Na scores; and ultimately to validate the model by a cohort of 94 patients with HBV-ACLF from three different geographical spread medical centers, so as to confirm the potential value of model for clinical treatment decision making.

## RESULTS

### Baseline characteristics of patients

305 patients who were diagnosed as HBV-ACLF (derivation cohort, n=211; validation cohort, n=94) were included in this study. The comparisons of patients’ characteristics in the derivation and validation cohorts were shown in Table 1. There was no significant difference in gender distribution, died numbers within 30-day, HE, ascites and laboratory parameters at baseline between derivation cohort and validation cohort. The pre-existing chronic liver disease (p<0.001), infection (p< 0.001) and PT (p=0.007) were significantly less in the validation cohort than those in the derivation cohort. The age (p=0.016) and suspicion of infection (p=0.005) were higher in the validation cohort than those in the derivation cohort.

**Table 1:** Clinical profiles of patients with HBV-ACLF in the derivation and validation cohorts

Parameters			Derivation cohort (n=211)	Validation cohort (n=94)	P values
Character	Age (years), mean±SD		43.94±12.63	47.15±10.60	0.016^*^
	Male, n (%)		176 (83.41)	81(86.17)	0.541
	Died within 30 days, n (%)		54 (25.59)	33 (35.11)	0.089
HE ^a^	None HE, n (%)		152 (72.04)	61 (64.89)	0.429
	I-II HE, n (%)		41 (19.43)	24 (25.53)	
	III-IV HE, n (%)		18 (8.53)	9 (9.57)	
Pre-existing chronic liver disease^b^	Chronic hepatitis B, n (%)		167(79.15)	49(52.13)	<0.001^*^
	Compensated liver cirrhosis, n (%)		44 (20.85)	45 (47.87)	
In-hospital complication	Ascites	None, n (%)	67 (31.75)	26 (27.66)	0.184
		Mild, n (%)	78 (36.97)	27 (28.72)	
		Moderate, n (%)	47 (22.27)	27 (28.72)	
		Severe, n (%)	19 (9.00)	14 (14.89)	
	Infection, n (%)		12 (5.68)	28 (29.78)	<0.001^*^
	Suspicion of infection, n(%)^c^		77 (36.49)	19 (20.21)	0.005^*^
Laboratory parameters at baseline	ALT (U/L), median (range)		163.50 (13.70 -2550.00)	197.75 (19.00 -7822.60)	0.227
	AST (U/L), median (range)		161.35 (31.50 -5715.00)	188.35 (21.00 -5688.20)	0.118
	TBil (μmol/L), median (range)		347.90 (171.93 -966.40)	325.75 (172.50 -780.30)	0.446
	INR, median (range)		2.55 (1.08 -5.04)	2.37 (1.32 -9.00)	0.127
	Na (mmol/L), median (range)		135.00 (111.00 -145.40)	135.00 (113.50 -143.70)	0.840
	Albumin(g/L), mean±SD		31.00±4.73	31.25±4.51	0.667
	WBC (×10^9^/L), median (range)		7.01 (1.10 - 29.41)	7.32 (1.11 -35.64)	0.489
	Blood neutrophils percentage count, median (range)		72.90 (26.60 -94.00)	70.90 (46.90 -92.50)	0.438
	Hemoglobin (g/L), mean±SD		119.63±23.74	121.04±25.99	0.642
	PLT (×10^9^/L), median (range)		87.00 (9.00 -298.00)	96.50 (26.00-297.00)	0.077
	PTA, median (range)		29.50 (8.00 -39.50)	32.00 (9.00 -40.00)	0.081

^a^ Mild to moderate hepatic encephalopathy (HE) was defined as grade I or II HE, severe HE was defined as grade III or IV HE, according to the West Haven classification.

^b^ The pre-existing chronic liver disease patients were divided into two groups, counter balanced between liver cirrhosis and chronic hepatitis B.

^c^ Suspicion of infection based on at least one of the following: WBC count >10,000/mm^3^ or ≥50% increase with respect to baseline with a final value >8,000/mm^3^; more than 5% of band forms; and/or temperature >37.5°C.

^*^ P<0.05.

### Predictors of mortality in the derivation cohort

30-day mortality was applied as the end-point in the multivariate logistic regression analysis, and all variables in univariate analyses were imported into the model. In this model, TBiL, albumin, HE, INR, blood neutrophils percentage count, and suspicion of infection were independent risk factors for death. The β-coefficient, OR and 95% CI for independent predictors were presented in Table 2.

**Table 2:** The initial model was based on independent predictors of mortality at admission.

Variables	OR	CI1	CI2	β-coefficient	P values
Ln (TBiL, μmol/L)	3.07	1.23	7.65	1.12	0.016^*^
Ln (Albumin, g/L)	0.98	0.91	1.06	-0.02	0.667
Ln (INR)	1.55	0.45	5.32	0.44	0.487
Ln (Blood neutrophils percentage count)	1.04	1.01	1.08	0.04	0.012^*^
HE					
I-II	1.16	0.49	2.75	0.15	0.735
III-IV	5.29	1.73	16.19	1.66	0.004^*^
Suspicion of infection^a^	0.30	0.14	0.68	-1.19	0.004^*^
Constant	0.00	0.00	0.01	-10.33	<0.001^*^

^a^ Suspicion of infection based on at least one of the following: WBC count >10,000/mm^3^ or≥50% increase with respect to baseline with a final value >8,000/mm^3^; more than 5% of band forms; and/or temperature >37.5 °C. ^*^P<0.05.

### The development of prognostic model

**Figure 1:**
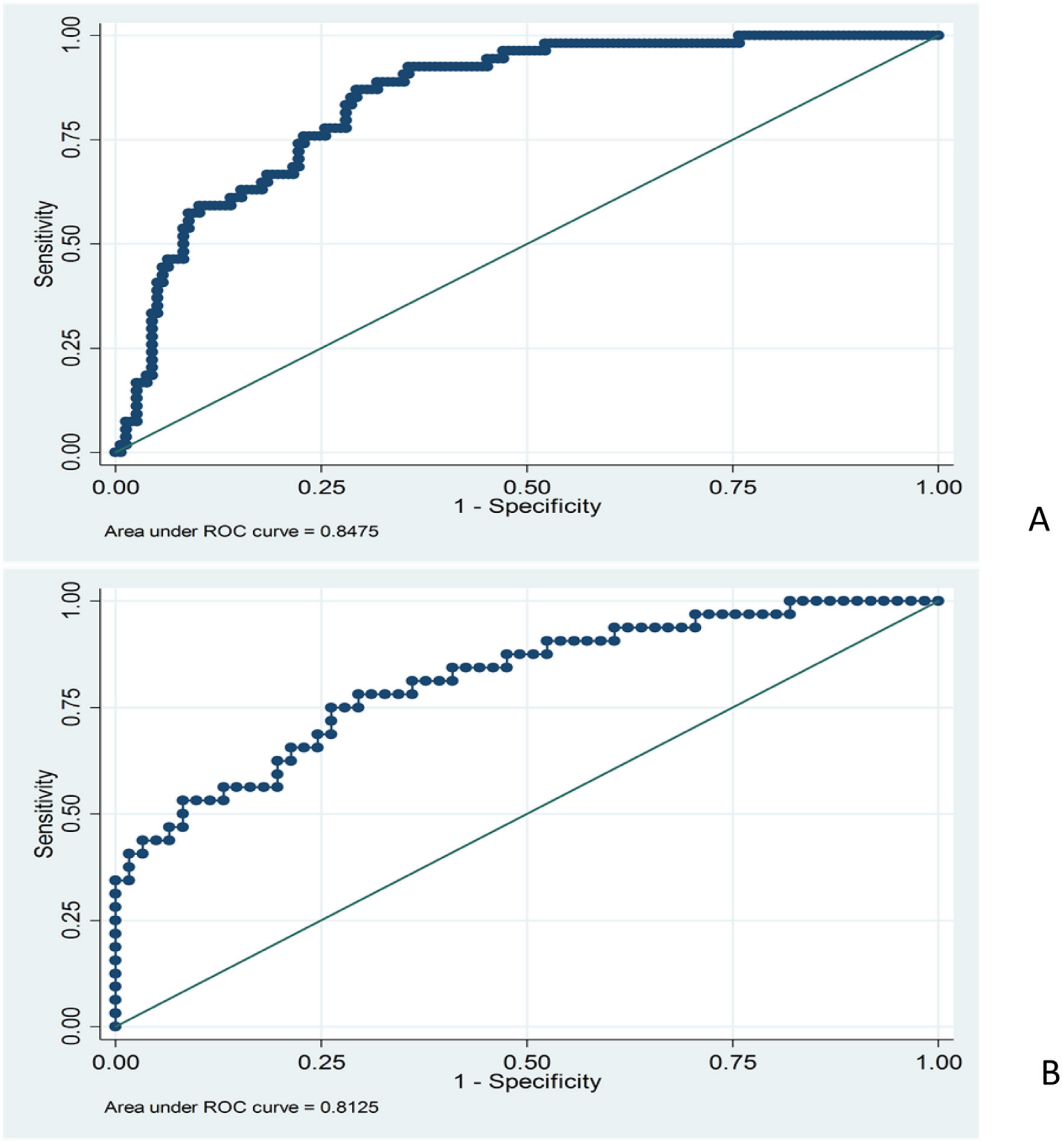
(A) Receiver operating characteristic curves of the HBV-ACLFD model in predicting mortality in the derivation cohort The HBV-ACLFD model had discrimination (AUROC=0.848; 95% CI: 0.793 to 0.902). **(B)** Receiver operating characteristic curves of the HBV-ACLFD model in predicting mortality in the validation cohort. The HBV-ACLFD model retained a good discrimination when applied to a validation cohort (AUROC=0.813; 95% CI: 0.720 to 0.905). The Hosmer-Lemeshow goodness-of-fit test for HBV-ACLFD model has been performed (p=0.612).

The prognostic model derived from the derivation cohort (Table 2). The initial model was established based on the independent predictors of admission mortality. The model passed the Hosmer-Lemeshow goodness-of-fit test (p=0.535); the area under the Receiver-operating characteristic (AUROC) curve was also used. However, its discriminative ability was only modest (AUROC 0.745, 95% CI: 0.667 to 0.823). Ultimately, the HBV-ACLF dynamic (HBV-ACLFD) model was constructed based on the independent predictors of mortality and the daily changes within 7 days after diagnosis (Table 3). The Hosmer-Lemeshow goodness-of-fit test for HBV-ACLFD model was also performed (p=0.288). The HBV-ACLFD model was discriminated (AUROC=0.848, 95% CI: 0.793 to 0.902; Figure 1A). The specificity, sensitivity, negative predictive value and positive predictive value of the HBV-ACLFD model were 93.63%, 44.44%, 83.05% and70.59%, respectively.

**Table 3:** The HBV-ACLFD model development based on predictors of mortality at baseline and their daily changes (Δ) within 7 days after diagnosis

Variables	OR	CI1	CI2	β-coefficient	P values
Ln (TBiL, μmol/L)	3.02	0.99	9.22	1.11	0.052
ΔLn (TBiL, μmol/L)	3928.33	1.71	9.02e+06	8.28	0.036^*^
Ln (Albumin, g/L)	0.93	0.84	1.02	-0.08	0.110
ΔLn (Albumin, g/L)	0.69	0.46	1.02	-0.38	0.063
Ln (INR)	2.25	0.56	9.05	0.81	0.255
ΔLn (INR)	9910.49	23.74	4.14e+06	9.20	0.003^*^
Ln (Blood neutrophils percentage count)	1.06	1.02	1.10	0.06	0.001^*^
ΔLn (Blood neutrophils percentage count)	1.38	1.13	1.68	0.32	0.002^*^
HE					
I-II	0.97	0.38	2.52	-0.03	0.957
III-IV	3.47	1.00	12.09	1.24	0.051
Suspicion of infection	0.37	0.15	0.87	-1.00	0.022^*^
Constant	0.00	0.00	0.02	-10.27	0.002^*^

^*^p<0.05.

### The validation of the predicted model

**Figure 2:**
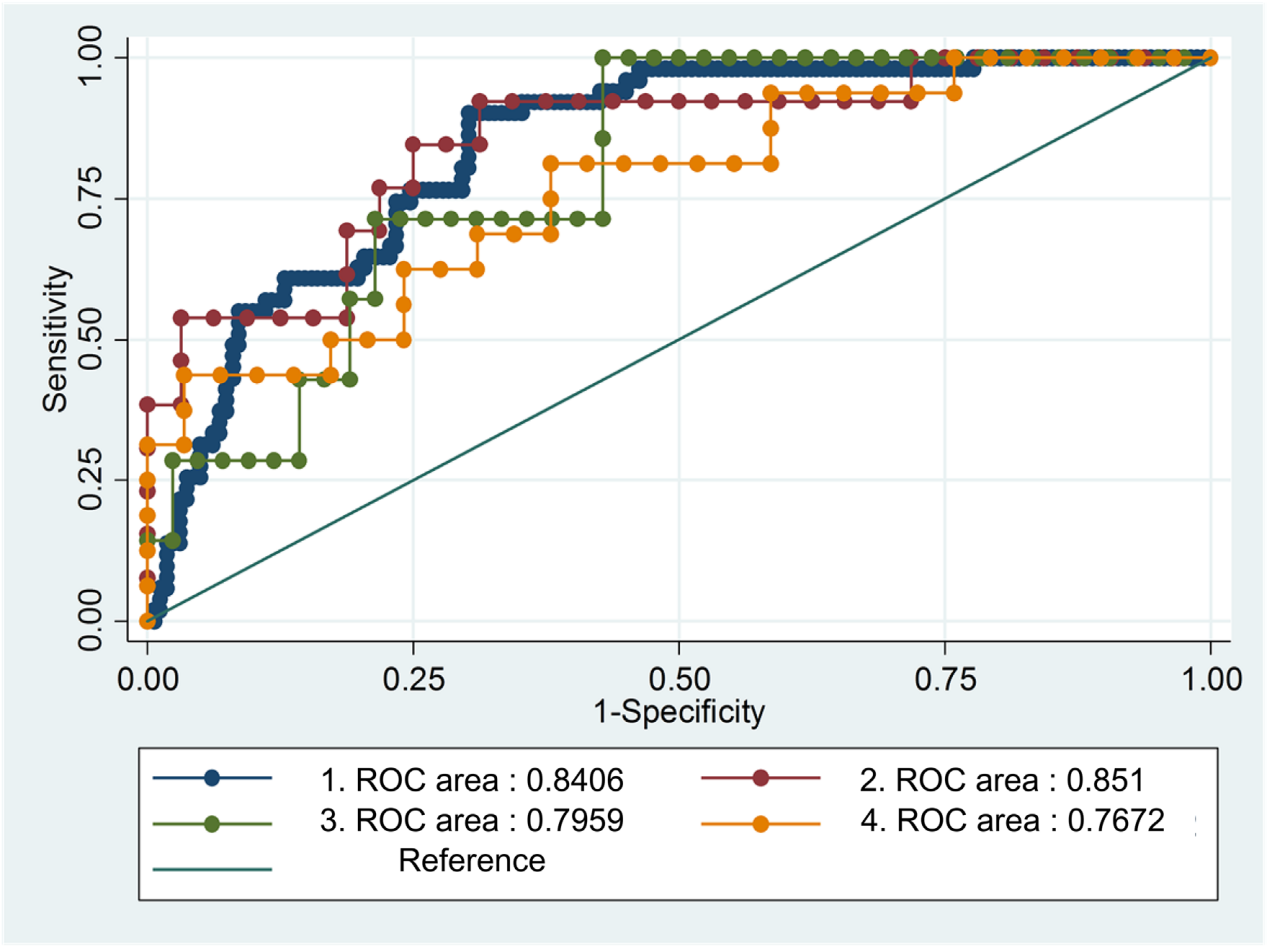
The AUROC from the three different medical centers 1: Beijing You-an Hospital (derivation cohort), 2: the Third Affiliated Hospital of Sun Yat-Sen University, 3: Tianjin Third Central Hospital, 4: Beijing You-an Hospital (validation cohort).

The HBV-ACLFD model retained a good discrimination when applied to a validation cohort (AUROC=0.813; 95% CI, 0.720 to 0.905; Figure 1B). The Hosmer-Lemeshow goodness-of-fit test for HBV-ACLFD model was also performed (p=0.612). The AUROC from three different medical centers were provided, respectively (Figure 2).

### Comparison with alternative predicted models

**Figure 3:**
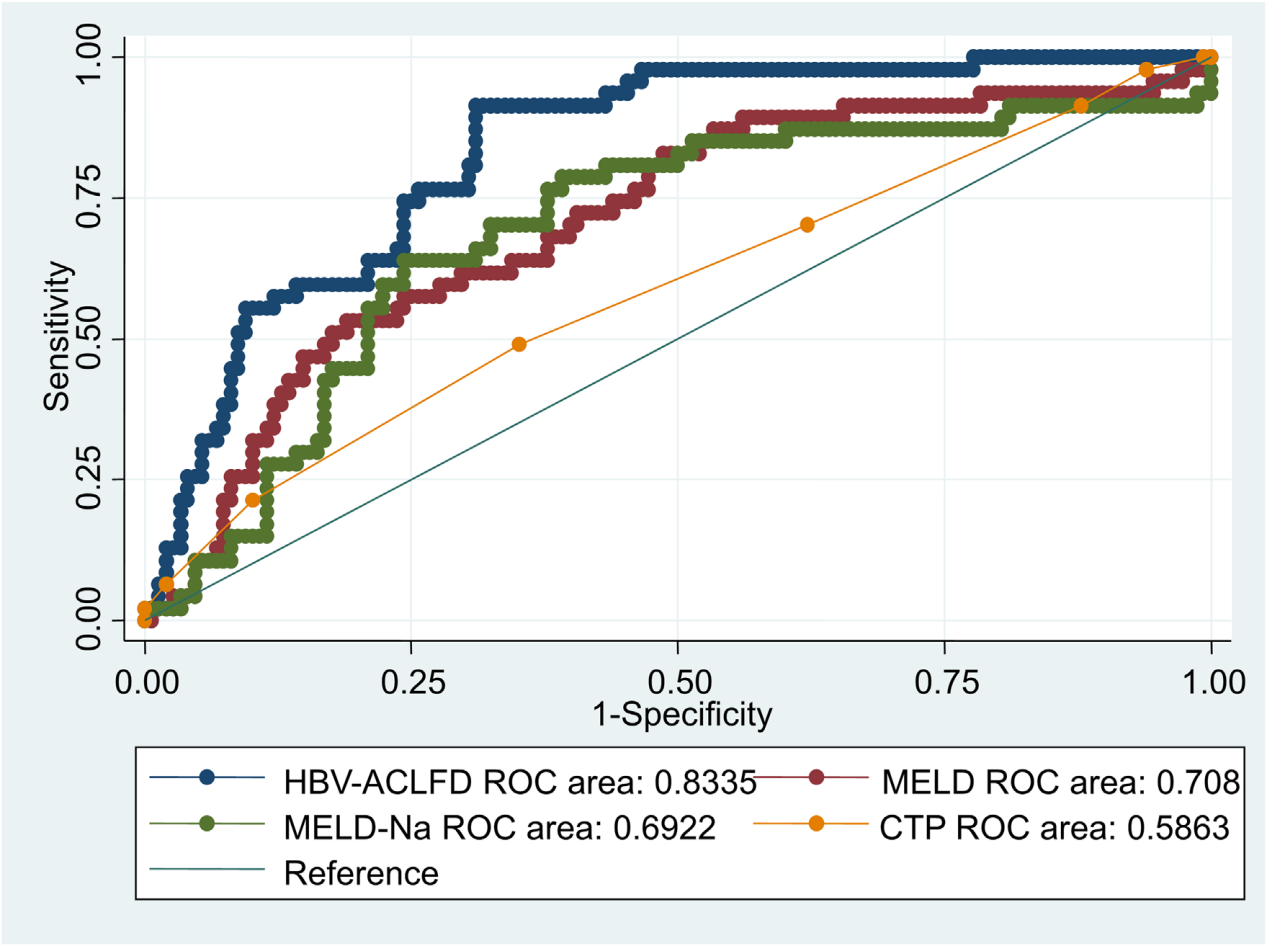
Comparison of the predictive accuracy for 30-day mortality between MELD, MELD-Na, CTP, and the dynamic prognostic model On admission, the AUROC was highest for the HBV-ACLFD model (0.848), followed by the MELD (0.696; 95% CI, 0.609 to 0.784), MELD-Na (0.686; 95% CI, 0.597 to 0.776), and CTP (0.566; 95% CI, 0.471 to 0.660) scores. There were significant differences between all pair of scores for the dynamic changes (p <0.05).

By the 30-day mortality and c-statistic as the endpoint, we identified four post-admission prognostic models of AUROC. The overall 30-day mortality was 25.59% (n=54 of 211) (Supplementary Figure 1). On admission, the AUROC was highest for the HBV-ACLFD model (0.848), followed by the MELD (0.696; 95% CI, 0.609 to 0.784), MELD-Na (0.686; 95% CI, 0.597 to 0.776), and CTP (0.566; 95% CI, 0.471 to 0.660) scores (Figure 3). There were significant differences between all pairs of scores for the dynamic changes (p <0.05).

## DISCUSSION

HBV-ACLF can cause irreversible liver failure, leading to severe liver function damage. If LT is not available, it may lead to an at least 70% mortality rate [15]. Therefore, it is urgent to guide and optimize therapeutic strategy for HBV-ACLF patients. An accurate prognostic scoring system is a precondition for guiding and optimizing therapeutic strategy for HBV-ACLF patients. The results of our research showed that the HBV-ACLFD model exhibited excellent discrimination and almost the same performance in two cohorts (AUROC 0.848 in the derivation cohort and 0.813 in the validation cohort). The performance of the HBV-ACLFD model appeared to be superior to MELD score, MELD-Na score and CTP score (P<0.0001). It is indicated that the HBV-ACLFD model can accurately predict 30-day mortality in patients with HBV-ACLF, which is helpful to select appropriate clinical procedures for HBV-ACLF patients, so as to relieve the social and economic burden.

A number of definitions of ACLF have been put forward, based on advice from experts rather than evidence-based data. The definitions of heterogeneity show the differences from the etiology of liver disease in Eastern and Western countries [12]. In China, chronic HBV infection contributes to the ACLF. The early diagnosis and prompt treatment play a core role in the liver failure therapeutic strategies. In the western countries, ACLF is defined on the basis of compensated or decompensated cirrhosis [3]. Acute liver function decompensation mainly caused by alcohol abuse and bacterial infection. The therapeutic strategies of western countries focus on the multiple organ dysfunction syndrome (MODS), to distinguish the patients with high mortality risk who need to be treated in the ICU or moved to the waiting list of LT [16]. In addition, there are many differences of pathophysiology, clinical manifestation and prognosis between HBV related liver disease and alcoholic liver disease. Actually, in the Asia-Pacific region, including China and India, ACLF is mainly induced by the HBV infection. Antiviral therapy is also the special treatment strategy in the comprehensive treatment of HBV related ACLF [12]. Therefore, it is necessary to build up an accurate prognostic scoring system based on HBV-ACLF patients specifically.

This study focuses on patients with HBV-ACLF from China. HBV-ACLF is one of the most lethal, costly, and pervasive diseases in China. Early predictors are essentially required to distinguish patients with ACLF who need orthotropic LT from those that can survive by only intensive medical care [17].

Currently, it remains difficult to identify appropriate indicators of poor outcome in HBV-ACLF [18]. The established prognostic models are mostly based on static baseline variables. However, patients’ responses to the treatment could also affect the outcomes [19]. Thus, we retrospectively reviewed patients diagnosed as HBV-ACLF. Patients with HBV-ACLF were divided into a derivation cohort and a validation cohort. The data of patients with HBV-ACLF, including demographics, clinical, laboratory variables, underlying chronic liver disease, complications during the hospital course, as well as in-hospital special treatment, were collected. The derivation cohort was used to identify predictors of 30-day mortality and construct the HBV-ACLFD prognostic model. Δbiomarker within 7 days after diagnosis was calculated and constructed into the model together with baseline risk factors based on logistic regression. The mortality rates in validation cohorts and derivation cohort with HBV-ACLF at 30-day after diagnosis were 25.59% and 35.11%, respectively, which are consistent with the results reported by Xia *et al* [20].

HBV-ACLF is a dynamic process in which the variables at the time of hospitalization are predicted to vary over time, accompanied with the clinical processes and outcomes change accordingly. Meanwhile, prognosis predictions fluctuate over different clinical treatment. A study of acute liver failure (ALF) has shown that the model based on the early change of dynamic variables is better for the prediction than the model based on static baseline variables [21]. The high absolute values of AFP cannot predict the prognosis well, but the uptrend of AFP over the first 3 days of hospitalization can reflect the survival rate of ALF [22]. Another study reported that ΔMELD is superior to initial MELD and that CTP scores are reliable in patients with advanced cirrhosis [14]. In our study, the initial model was constructed based on the independent predictors of admission mortality. However, the discriminative ability was just moderate (AUROC = 0.7451). When Δbiomarker was calculated and constructed into the model together with baseline risk factors, this model had a great discrimination (AUROC=0.8475). Taken together, it is suggested that continuous changes in predictive variables can better predict mortality than using static variables.

On admission, the AUROC for MELD, MELD-Na, and CTP scores were 0.6962, 0.6862, 0.5656, respectively, which is consistent with the results reported [23]. However, the MELD, MELD-Na and CTP scoring systems are used to predict mortality risk in untreated cirrhotic patients [24–25]. These models did not consider the impact of biomarker changes after diagnosis, HBV-ACLF related complications and in-hospital special treatment on the prognosis. When HBV-ACLF related complications and biomarker (Δbiomarker) changes daily within 7 days after diagnosis were established with baseline risk factors according to logistic regression, the HBV-ACLFD model (AUROC=0.8475) showed better potential than the established prognostic models like MELD, MELD-Na and CTP scores in a cohort of patients with HBV-ACLF for predicting the 30-day mortality. By the AUROC, these results support that Δbiomarker and HE can improve its accuracy in predicting mortality. Thus, the HBV-ACLFD model showed good discrimination in the derivation cohort. When applied to the separate validation cohort of patients with HBV-ACLF, the new model retained good discrimination, accurately distinguishing the ACLF patients who need LT, and grasping the best opportunity for their transplantation.

We also evaluated biochemical and clinical variables via multivariate logistic regression. HE, suspicion of infection, baseline and average daily changes of serum TBiL, INR, serum albumin and blood neutrophils percentage were independent prognostic factors for 30-day mortality. Meanwhile, on multivariate analysis, suspicion of infection is associated with mortality. The hazard ratio for mortality of patients with HBV-ACLF was 30.37 during the first 30-day. It is recommended that patients with high risk of complications and cirrhosis are highly suspected bacterial infection [26].

The systemic inflammatory response syndrome (SIRS) occurs in patients with advanced cirrhosis and is correlated with poor prognosis [27]. The definition of SIRS and sepsis are very difficult because of the following findings [28]: elevated baseline heart rate for hyperdynamic circulatory syndrome; reduced baseline cell count for hypersplenism; hepatic encephalopathy leading to excessive ventilation; cirrhotic patients with mildly elevated body temperature [26]. The suspicion of infection is correlated with a good prognosis [29]. Rapid initiation of appropriate antibiotic therapy is critical to the management of patients suspected of being infected. Delayed and inappropriate treatments are correlated with increased mortality [30].

Moreover, the validation cohort data were collected from three different medical centers of geographic location in China, which means our model is validated in different regions of the population and in different areas of HBV-ACLF lesions. It also improves the accuracy and credibility of our HBV-ACLFD model, which is another principal strength of our model. Because this model is based on retrospective data on a great number of patients with HBV-ACLF from three different medical centers, it also means that homogeneous cohort managed with similar treatment regimens cannot be secured. It is indicated that this model has a broad spectrum of practicality.

However, novel clinical strategies, including artificial liver support system and stem cell transplantation, apply to treat HBV-ACLF now. Our model cannot assess the effect of these novel clinical strategies for accurately predict outcome in patients with HBV-ACLF. Meanwhile, the comparison between the CLIF Consortium ACLF score (CLIF-C ACLFs) and the HBV-ACLFD model should be explored, despite the fact that the CLIF-C ACLFs is helpful to predicting short-term mortality in ACLF patients in Western countries [31–32], where the most common etiology of ACLF is alcoholic liver disease.

In conclusion, we retrospectively deduced and validated the dynamic models of predictive outcomes in patients with HBV-ACLF. This model may be helpful in clinical decision making and risk stratification for patients with HBV-ACLF.

## MATERIALS AND METHODS

All procedures and methods related to this research were accorded morally with current laws as well as the creeds of the Declaration of Helsinki. The research was permitted by the Ethical Committee of Beijing You-An Hospital, Capital Medical University.

### Study design and patients selection

A total of 445 patients who were diagnosed as HBV-ACLF from January 2005 to February 2014 were included in this research. The minimum follow-up period for enrolled patients was 30-day after diagnosis. The diagnosis of cirrhosis was according to a composite of clinical signs and findings provided through laboratory test results, radiologic imaging, endoscopy and liver biopsy.

The entry criteria comprised the following:HBV-ACLF is defined as ACLF with previously diagnosed or undiagnosed HBV. All enrolled patients met the criteria for ACLF from the consensus recommendations of the Asian Pacific Association for the Study of the Liver (APASL) [33]. All treatments were performed based on the criteria of diagnostic and treatment guidelines for ACLF adopted by the Chinese Medical Association [34].

**Figure 4:**
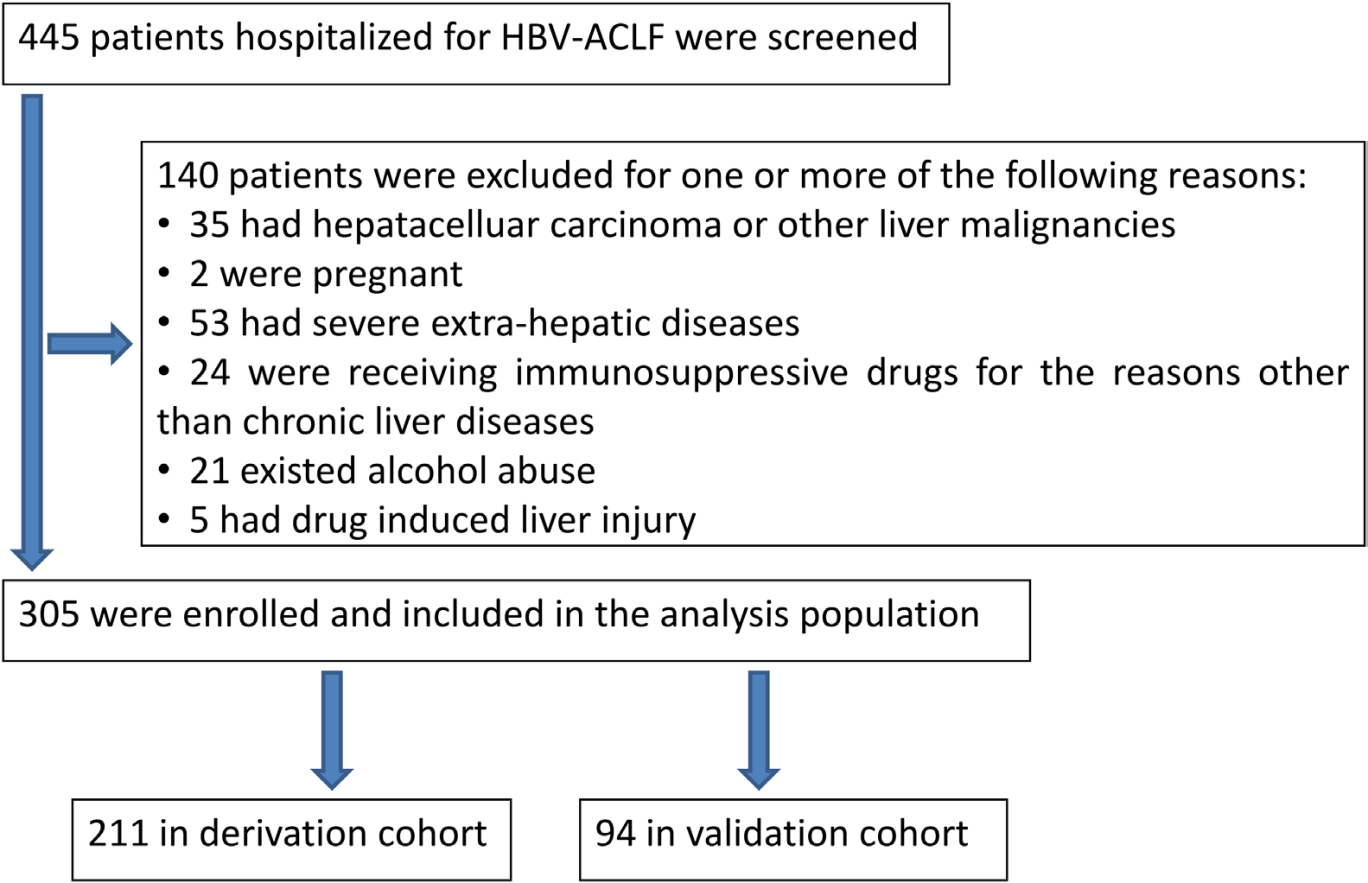
Study flow: diagram showing the process of study selection and exclusion in of HBV-ACLF patients

The exclusion criteria were the following: other factors induce severe liver injury, such as alcohol, drugs, hepatoviruses other than HBV, autoimmunity and pregnancy, as well as genetic and metabolic disorders. HBV-ACLF patients with hepatocellular carcinoma, known decompensated cirrhosis prior to onset of acute hepatic insult, age less than 18 years, jaundice induced by hemolytic jaundice and obstructive jaundice, absence of any chronic liver disease on investigations, and prolonged prothrombin time induced by blood system diseases were also excluded. The flowchart for the selection of HBV-ACLF patients was shown in Figure 4.

Derivation cohort data were screened from Beijing You-an Hospital from January 2005 to January 2013, while validation cohort data was collected from three different medical centers from January 2013 to February 2014. Specifically, 30 cases, 24 cases and 40 cases of HBV-ACLF as validation cohort data were collected from Beijing You-an Hospital, Tianjin Third Central Hospital and the Third Affiliated Hospital of Sun Yat-Sen University, respectively. The derivation cohort was applied to determine the predictors of mortality and thus established a prognostic model.

### Observed parameters

Data on patient demographics, clinical and laboratory variables, evaluation of the underlying chronic liver disease, complications during the medication course, in-hospital special treatment (antiviral therapy, plasma exchange, corticosteroid exposure) were retrospectively collected. All enrolled patients were followed up for a minimum of 30-day by clinic visits or telephone. The outcome (death or survival) of each patient with HBV-ACLF was documented. Suspicion of infection based on at least one of the following: WBC count >10,000/mm3 or ≥50% increase with respect to baseline with a final value >8,000/mm3; more than 5% of band forms; and/or temperature >37.5°C.

The results of blood tests performed on the day of diagnosis and within 7 days after diagnosis were recorded. The blood tests consisted of white blood count (WBC), platelet count (PLT), hemoglobin level, blood neutrophils percentage count (NEUT), international normalized ratio (INR), prothrombin time (PT), creatinine level, aspartate transaminase (AST) level, aspartate alanine transaminase (ALT) level, TBil level (total bilirubin), direct bilirubin (DBil) level, cholinesterase, glucose, total cholesterol level, albumin level, triglyceride level, serum sodium level, ammonia level, serum chloride level, serum potassium level, serum magnesium level and total serum calcium level.

Differences of biomarker (Δ biomarker) levels within 7 days after diagnosis were calculated. For example, a patient with HBV-ACLF with an ALT of 100U/L on the day of admission and 380U/L on day 7 after admission, had ΔALT= (380-100) /7=40. In the case of variceal hemorrhage or plasma exchange, the results of blood tests after variceal hemorrhage or plasma exchange were chosen after more than 2 days.

### Management protocol

Patients who were positive for HBV-DNA at presentation underwent antiviral therapy (lamivudine, telbivudine, or entecavir) with informed consent. In order to prevent/treat complications, comprehensive medical interventions were applied, including absolute bed rest, intravenous drop infusion of albumin or plasma, energy supplements, maintenance of acid-base equilibrium or electrolyte, plasma exchange the use of adenosylmethionine, glutathione or branched-chain amino acids to nourish liver cells, as well as antibiotics for infection. Patients with decompensation requiring organ support (such as variceal hemorrhage, HE, hepatorenal syndrome, mechanical ventilationor multiorgan failure) were admitted to ICU.

### Calculation of the CTP, MELD and MELD-Na

The MELD equation was applied to calculate the score of severity: 9.57×ln(creatinine, mg/dl)+3.78×ln(bilirubin, mg/dl)+11.2×ln(INR)+6.43, in which the minimal values were forced to 1.0 for calculation purposes [29]. The MELD-Na equation was constructed based on the Na and MELD, MELD+1.59×(135–Na), with minimum and maximum Na values as 120 and 135 mEq/L, respectively [35]. The CTP classification was assessed according to the standard criteria [36].

### Procedures

The derivation cohort was applied to determine the predictors of mortality and thus established a prognostic model. Δbiomarker was calculated and constructed into the model together with baseline risk factors based on logistic regression, which is our dynamic prognostic model for HBV-ACLF, named the HBV-ACLF dynamic (HBV-ACLFD) model. AUROC were used as a control to compare the predictive values in the HBV-ACLFD model, i.e. MELD score, MELD-Na score and CTP score.

### Statistical analysis

All statistical analyses were conducted by STATA version 13.1. Univariate analyses were applied by appropriate tests to identify the variables which were significantly different in patients who died or survived in the derivation cohort. Multivariable logistic regression model was used by taking these predictor variables with the outcome (survived vs. death) through a stepwise forward selection procedure and thereby establish the dynamic prognostic model. The validity of this model was constructed by concordance (c) statistics, which is equivalent to the area under the AUROC curve. C-value >0.7 was recommended useful, and the C - value >0.8 would be considered excellent. Then, the performance of this model was finally verified in an independent cohort.

## SUPPLEMENTARY MATERIALS FIGURE


