# Supplementary material for: A novel dynamic model for predicting outcome in patients with hepatitis B virus related acute-on-chronic liver failure
Source: Oncotarget. 2017 Nov 14;8(65):108970–80. doi: 10.18632/oncotarget.22447 (PMC5752496; doi:10.18632/oncotarget.22447)
Supplement: Supplementary file 1 [file oncotarget-08-108970-s001.pdf]

A novel dynamic model for predicting outcome in patients with hepatitis B virus related acute-on-chronic liver failure

SUPPLEMENTARY MATERIALS

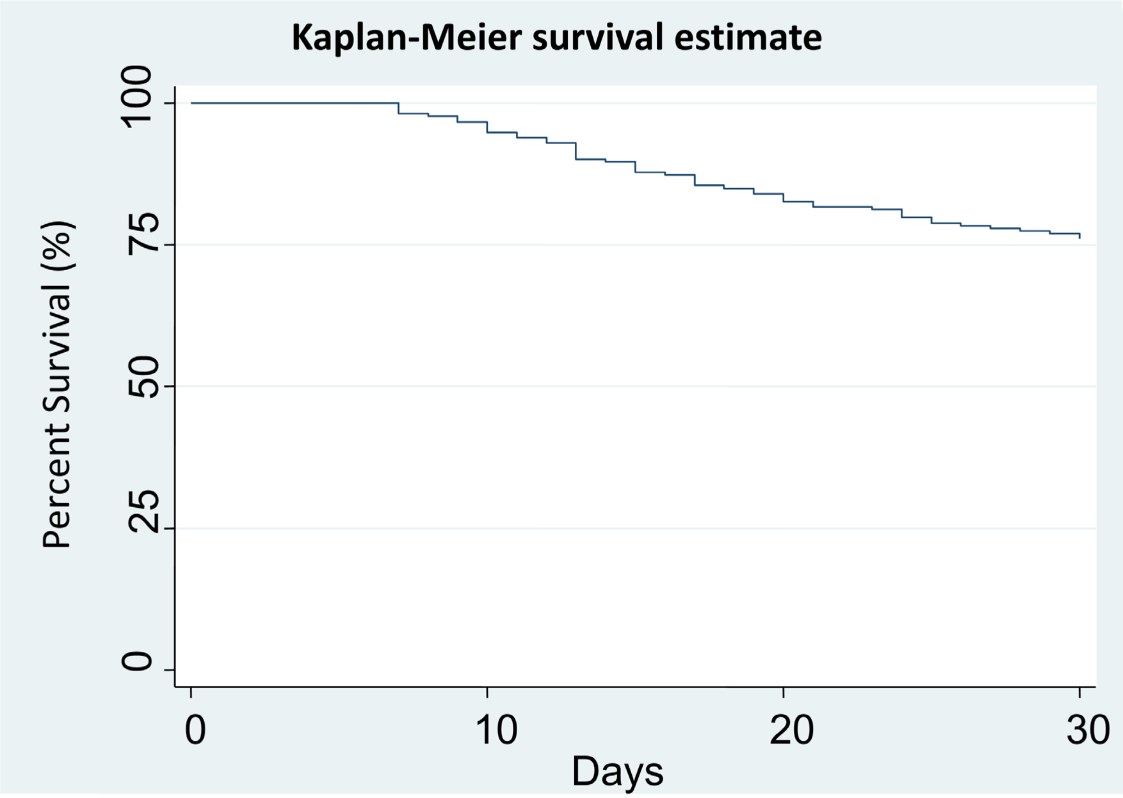

Supplementary Figure 1: Overall survival curves of 211 patients with HBV-ACLF in the derivation cohort. The survival curve was calculated according to the Kaplan-Meier method.
